# Footprinting of Inhibitor Interactions of *In Silico* Identified Inhibitors of Trypanothione Reductase of *Leishmania* Parasite

**DOI:** 10.1100/2012/963658

**Published:** 2012-04-01

**Authors:** Santhosh K. Venkatesan, Vikash Kumar Dubey

**Affiliations:** Department of Biotechnology, Indian Institute of Technology Guwahati, Assam 781039, India

## Abstract

Structure-based virtual screening of NCI Diversity set II compounds was performed to indentify novel inhibitor scaffolds of trypanothione reductase (TR) from *Leishmania infantum*. The top 50 ranked hits were clustered using the AuPoSOM tool. Majority of the top-ranked compounds were Tricyclic. Clustering of hits yielded four major clusters each comprising varying number of subclusters differing in their mode of binding and orientation in the active site. Moreover, for the first time, we report selected alkaloids and dibenzothiazepines as inhibitors of *Leishmania infantum* TR. The mode of binding observed among the clusters also potentiates the probable *in vitro* inhibition kinetics and aids in defining key interaction which might contribute to the inhibition of enzymatic reduction of T[S] 2. The method provides scope for automation and integration into the virtual screening process employing docking softwares, for clustering the small molecule inhibitors based upon protein-ligand interactions.

## 1. Introduction

Leishmaniasis is a protozoan disease caused by the parasite belonging to the genus *Leishmania*; the disease belongs to group of neglected tropical diseases as defined by World Health Organization. The disease is caused by 20 different species belonging to the genus *Leishmania*. The causative species of visceral leishmaniasis (VL) include *Leishmania donovani, Leishmania infantum* in Asia, Africa, and Europe (Old World), and *Leishmania chagasi* in South America (New World) [1–4]. Trypanothione metabolisms involving various enzymes including Trypanothione reductase which has been ideal target for designing chemotherapeutics [5–7]. The absence of TR in humans makes it an attractive target for rational drug design towards Leishmaniasis.

Only a very limited number of drugs have been developed for the treatment of Leishmaniasis over the past 60 years, and the use of available drugs has been hampered by high cost, adverse side effects, development of resistance by the parasite, and also the efficacy [8]. Some experimental as well as *in silico *attempts have been made to identify inhibitors or subversive substrates of TR [9, 10]. TR is a homodimer, and the active site residues are contributed by both the chains, and docking and crystalisation studies on TR of *Trypanosoma cruzi* with tricyclic compounds has shown that they bind to the hydrophobic wall on active site formed by Trp21 and Met113 [11, 12], but in case of *Leishmania infantum,* trypanothione reductase docking studies show that it binds to the hydrophobic region formed by Phe396, Leu399, and Pro462 [13]. TR active site is negatively charged with surrounding hydrophobic residues, while GR of mammalian counterpart is positively charged. Thus, a typical specific inhibitor of TR should have an extended hydrophobic region and an overall positive charge, where charge plays a major role in binding of the inhibitor to the active site and also in discrimination between a TR and GR inhibitor [14]. The additional hydrophobic region present in proximity of the active site was formed by residues Phe396, Pro398, and Leu399. The conservative substitution of these in TR by Met406, Tyr407, Ala409 in human GR and can be rationally explored to design inhibitors specific towards parasite TR.

There is an urgent need for efficient antileishmanial chemotherapeutic agents, with the advent of automated computational techniques; we aim to identify novel TR inhibitors which can be potential antileishmanial agents. Structure based drug design (SBDD) has gained importance over the last few years, due to its potential to identify novel lead compounds in the drug designing process. SBDD comprises two broad computational categories, they are based upon the protein-ligand interactions, ligand similarity searches [10]. Methods using protein-ligand interactions employ docking in their screening process, and pharmacophore generation is performed in case of ligand similarity searches. Virtual screening of small molecule databases is now a well-established protocol for identification of potential lead compounds in the drug designing process, provided the three-dimensional structure of the protein is known. Structure-based virtual screening approach is primarily applied as a hit identification tool and also used in lead optimization; the aim is to reduce a large number of compounds to a smaller subset which can be biologically active against the target. The process of virtual screening to design inhibitors towards an enzyme involves modeling of the binding site of the inhibitor at the active site of the enzyme through docking procedures and scoring, ranking of those compounds to narrow down to a smaller subset which contains potential biologically active inhibitors [15, 16].

In our study, NCI Diversity set II was used as small molecule chemical library owing to the diversity of chemical entities present in the set, and for small molecule conformational search AutoDock4 [17], molecular docking program was performed. Based upon the binding energies, the highest ranked structures from the docking program were clustered to ligand-foot-print the interactions of diverse compound sets aiding in classification of differential binding modes exhibited by small molecules at the active site of TR. The interactions were clustered from protein-ligand complexes using AuPosSOM [18], and they were also classified into subgroups. Four different major clusters were obtained based upon the interaction of inhibitors on the active site of TR; each cluster exhibiting differences in the mode of binding and subclusters within clusters showed conservation in their binding pattern. The inhibitors bind primarily to the hydrophobic stretch formed by Leu399 which is in close proximity to the active site commonly known as the Z-site. *In silico* studies on other drug targets proteins are also ongoing in our laboratory [19].

## 2. Methods

### 2.1. NCI Diversity Set II

The National Cancer Institute Diversity set II (http://dtp.nci.nih.gov/branches/dscb/diversity_explanation.html) is a structural database selected from NCI chemical library. The webpage also provides details of compounds like molecular weight and so forth; 2D SDF data set of the compounds available online was downloaded and used for generation of three dimensional structure coordinates of small molecules using ChemDraw 3D ultra 8.0 software (Molecular Modelling and analysis; Cambridge soft Corporation, USA (2003)).

### 2.2. Ligand and Protein Preparation

The NCI Diversity set II 2D SDF files were obtained, they were submitted to Online SMILES Translator to obtain three dimensional co-ordinates, the multi-PDB file was split and converted into PDBQT format, input format for AutoDock4.The charges on the ligand atoms were preserved, nonpolar hydrogens were merged, default rotatable bonds were retained using TORSDOF utility [20]. The crystal structure of *Leishmania infantum* TR (PDB ID: 2JK6) resolved at 2.95 Å was retrieved from the Protein Data Bank [21]. Affinity grids of size 80 × 80 × 80 Å with 0.200 Å grid spacing around the active site, affinity grid maps were generated for each of the atom types present in the protein and all possible atom types (HD, C, A, N, NA, OA, F, P, SA, S, Cl, Br, and I) in the NCI Diversity set II. An electrostatic and a desolvation grid map were also generated.

### 2.3. Docking

Docking simulations were performed as described earlier using Lamarckian genetic algorithm (LGA) [13]. LGA is a hybrid of genetic algorithm, and local search algorithm the other two algorithms available with AutoDock4 [16, 22, 23].

The active site of TR was kept rigid, and nonflexible docking was carried out. The docking parameters for the virtual screening process were set to default with the exception of the following: trials of 20 Lamarckian Genetic Algorithm runs with each case (ga_run, 20), initial population size of 300 (ga_pop_size, 300), random starting position and conformation, and 250,000 (ga_num_evals, 250,000) energy evaluations. Each docking simulation produced 20 different docked conformations, which were then clustered based upon Root-Mean-Square Deviation (RMSD) of the different bound conformations; the RMSD difference between conformations within each cluster will be less than 2 Å. The binding energy of each cluster is the mean binding energy of all the conformations present within the cluster; the cluster with lowest binding energy and higher number of conformations within it was selected as the docked pose of that particular ligand.

### 2.4. Preparation of Protein-Ligand Complexes and Clustering

Protein-ligand complexes were written for the top-ranked compounds obtained after sorting the results obtained from the multiple docking simulations; they were sorted based upon their binding energy and also on the basis of number of conformations per cluster. Clustering of the protein-ligand complexes was performed to classify the protein-ligand interaction; AuPosSOM (Automatic analysis of poses using SOM) was used for this purpose [18]. The clustering of the protein-ligand complexes is primarily carried out in three steps. A self-organising map (Kohonen SOM) training is initially performed to define protein-ligand contact descriptors, based upon the ligand interaction footprints, the complexes are clustered, and for visualisation purpose, a Newick tree file is generated. Interleaved vectors are generated for all the atoms in the protein-ligand complexes. Vector generation is done using BioPython scripts; this takes into consideration both bonding and non-bonding interactions possible in the protein-ligand complex. The generated are then trained using Kohenen's self-organising maps; the trained vectors are then clustered. The SOM generate was used for construction of Newick tree, which was prepared using Dendroscope. Figures showing bonding and nonbonding interactions were prepared using LIGPLOT program [23]; figures graphically representing the mode of interaction were prepared using UCSF Chimera.

## 3. Result and Discussion

**Figure 1:**
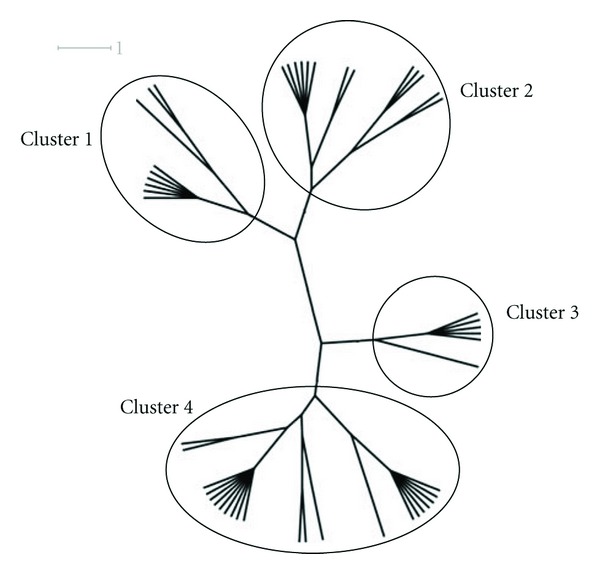
Cluster tree showing four major clusters (Cluster 1, Cluster 2, Cluster 3, and Cluster 4), each cluster signifies a different ligand footprint on the protein-ligand complex. The protein-ligand interactions within the cluster are conserved; they vary between the clusters. The subclusters within a cluster also show similar interaction modes with slight variation in the binding pattern.

**Figure 2:**
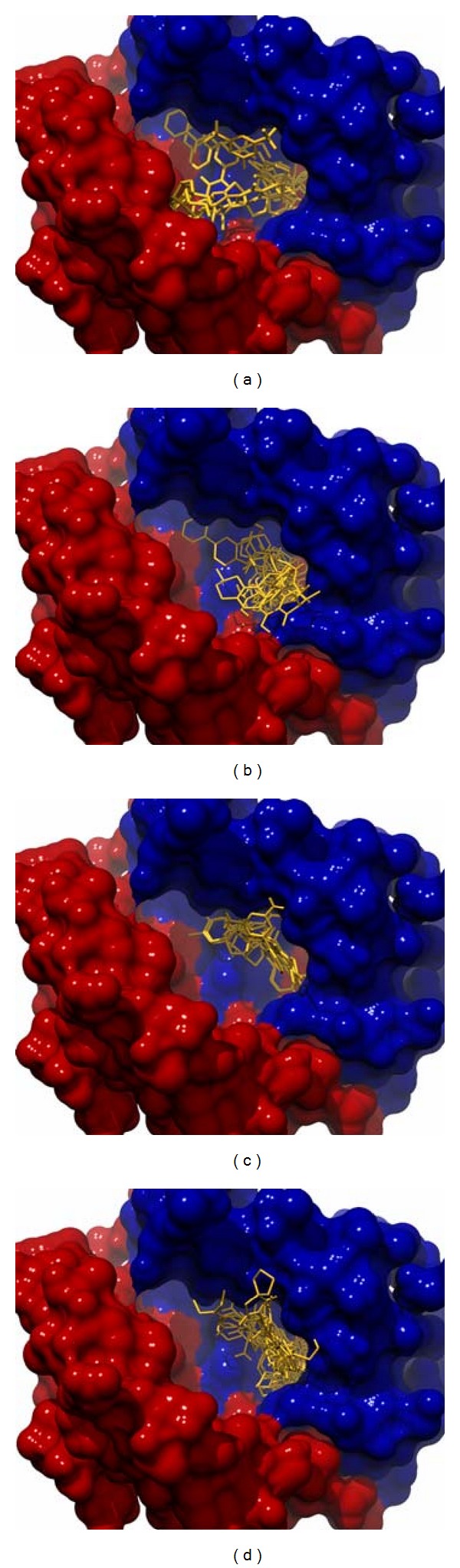
Orientation of inhibitors segregated into four different clusters at the active site of TR. Inhibitors in Cluster 1 (a) position themselves between both the hydrophobic patches of the active site, Cluster 2 (b) bind to the Z site with their side chains orienting themselves towards the negatively charged residues of the active site. In Cluster 3 (c), the ligands primarily bind to the negatively charged residues such as Glu466, Glu467 residues involved in orientation of active site histidine during hydride transfer process, in Cluster 4 (d) comprise of compounds which stack to the Z site, in the vicinity of the active site and they also interact with Lys61 of the substrate-binding site.

The results obtained from the virtual screening process were sorted, and they were ranked based upon their binding energy; from the diversity set, top 50 ranked structures were used for further clustering analysis (Table 1), and the criteria set to identify the binding pose were lowest binding energy, maximum number of conformations in the cluster. Clustering was performed downstream of virtual screening to classify the inhibitor scaffolds by contact-based analysis, clustering with AuPosSOM gave four different major clusters and each major cluster had subclusters except Cluster 3 (Figure 1). Each major signifies a differential binding mode of compounds confined within that cluster; both hydrogen bonding and non-bonding interaction were taken into consideration for contact-based clustering of protein-ligand complexes. A donor hydrogen list and acceptor list is generated by AuPosSOM to calculate potential hydrogen bonds, when a hydrogen bond donor and an acceptor are present within 3 Å, then it is taken into consideration as a hydrogen bond. As listed in Table 2, protein-ligand interactions within a cluster were greatly conserved, whereas sub clusters within each cluster showed slight degree of variation in the observed mode of interaction (Figure 2).

### 3.1. Interaction of Compounds with Conventional Hydrophobic Patch of Active Site

In cluster 1, the ligands traverse the active site region and have hydrogen bonding potentials with amino acids of both conventional hydrophobic wall and also the Z site residues. The cluster contained two sub-clusters (SC) with eight (SC-1) and three (SC-2) in each. Inhibitors belonging to this cluster are in hydrophobic interaction with amino acids such as Tyr110, Trp21, Glu18, and Met113, where Tyr110 is a key residue aiding in anchoring of T[S]_2_ towards the hydride transfer region, Glu18 provides a negative charge to the active site, and Trp21 and Met113 form a nonpolar patch in the substrate-binding site of TR where spermidine moiety of T[S]_2_ would be located in the rest of the amino acids which are in hydrophobic interaction to the core of the conventional hydrophobic wall providing substrate specificity to the TR active site. **Compound 21** of SC-2 produced 20 different conformations out of genetic algorithm runs performed, which can be attributed to the higher number of torsions in the ligand, making it a diverse binding compound.

**Figure 3:**
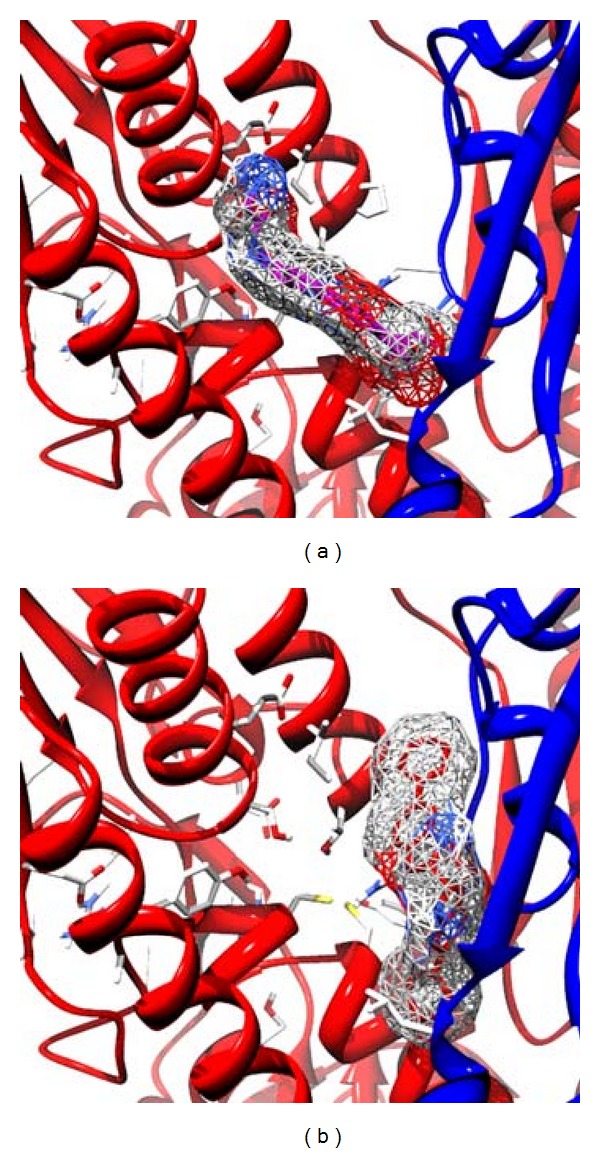
Compound 16 (a) of cluster 1 traversed between hydrophobic patches formed by Trp21, Met114 and Leu399, Pro492, and Pro398 where later is located in vicinity to the substrate-binding site, it also showed hydrogen bonding potential with Tyr110 of the active site, Tyr110 anchors the substrate to the active site by hydrogen bonding with the spermidine moiety. Compound 48 (b) of Cluster 2 binds to the Z site with hydrogen bonding potentials with His461, active site histidine base, and also with the residues which aid in orientation of substrate towards the active site.

**Figure 4:**
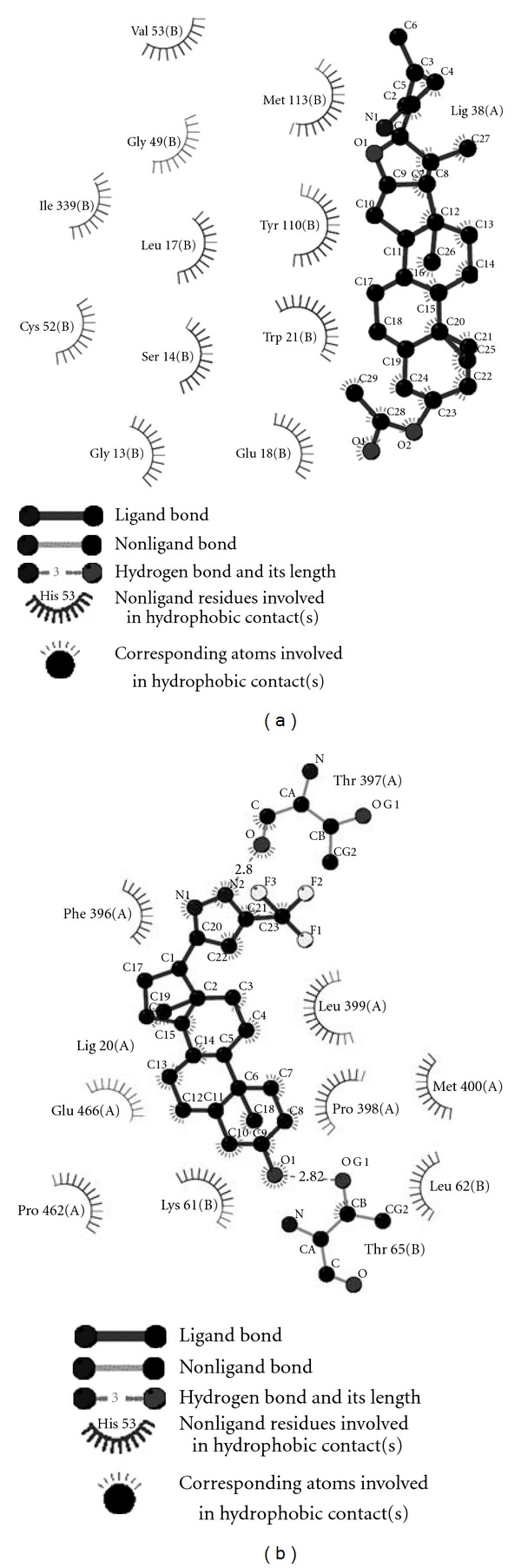
Ligplot showing proposed hydrogen bonding and non bonding interaction of Compound 38 (a) and Compound 20 (b) from Cluster 1, Cluster 2, respectively. They bind to the active site with specific interaction pattern representation of each cluster. Compound 38 stacks itself between two hydrophobic patches in the active site, whereas compound 20 is in nonbonding interaction with Z site residues and Glu of active site.

In SC-1 **Compound 16 **(Figure 3(a)) formed hydrogen bonding with Tyr110 and Glu18 which are key residues in the active site providing substrate specificity and anchoring substrate to the active site, respectively; as a conserved pattern among the cluster 1 compounds, this compound also had hydrophobic interactions with residues of both Z-site and amino acids providing net negative charge key for lodging of T[S]_ 2_ to the active site. The higher affinity of this compound to the active site was due to hydrophobic interaction of it with hydrophobic patch formed by Trp21, Met113, and Cys52, His461 (active site histidine base) of the hydride transfer region, whereas **Compound 38 **(Figure 4(a)) had hydrophobic interaction with all the residues in the core hydrophobic patch of the active site. Most compounds present within this cluster were linear pentacyclic compounds occupying a higher steric space in the active site and thus potentially can inhibit the reduction of T[S]_ 2_ by competing for active site binding region. **Compound 8**, an alkaloid named tomatidine (a natural compound from Solanum Spp), also showed interactions of a potential inhibitor with the same hydrophobic and hydrogen bonding interactions that are observed within this cluster, potentiating them as scaffolds to study as inhibitors of TR. In SC-2, the conserved pattern was along with hydrophobic interactions with that of the conventional hydrophobic patch, but the cyclic structures also bind to the negatively charged region in the active site comprising Glu466, Glu467, and **Compound 5** belonging to this subcluster formed hydrogen bonding with Lys61 which is in close proximity to the active site.

### 3.2. Binding of Inhibitors in Vicinity of Hydride Transfer Region

In cluster 2, there were four sub-clusters (SC3-SC6), the conserved interaction observed among this cluster was the inhibitors are in hydrophobic interaction with amino acids His46, Thr65, and the Z site residues, where His461 forms the core of the hydride transfer region along with Cys52, Cys57. The inhibitors belonging to this cluster may potentially inhibit the reduction of T[S]_ 2_, by disrupting the hydride transfer from His461 to FAD of the active site and then to active site Cystines, by being in hydrophobic interaction and hydrogen bonding interaction with the key residues of hydride transfer process. Different bound conformation was attained with every run in case of **Compound 26** and **Compound 7** in SC-3, making them a differentially binding compound with an RMSD of more than 2 Å between each bound conformation generated by the docking process.

As representative from this cluster, the binding of **Compound 48 **(Figure 4(a)) belonging SC-5 to the active site is depicted in Figure 3(b). The compound binds to the active site having hydrophobic interactions with residues from the Z site hydrophobic patch and also having hydrogen bond forming potentials with residues involved in hydride transfer; this was the conserved mode of interaction observed within this sub-cluster, in case of **Compound 20 **of SC-3 (Figure 4(b)), the compound formed hydrogen bonds with Thr397 and Thr65 which are in vicinity of the active site residues. The cyclic structures of the compound stack themselves between the hydrophobic patch formed by the Z-site residues and the negatively charged region comprising Glu466 and Glu467.

### 3.3. Interaction of Inhibitors with Glu466, Glu467 of the Active Site

**Figure 5:**
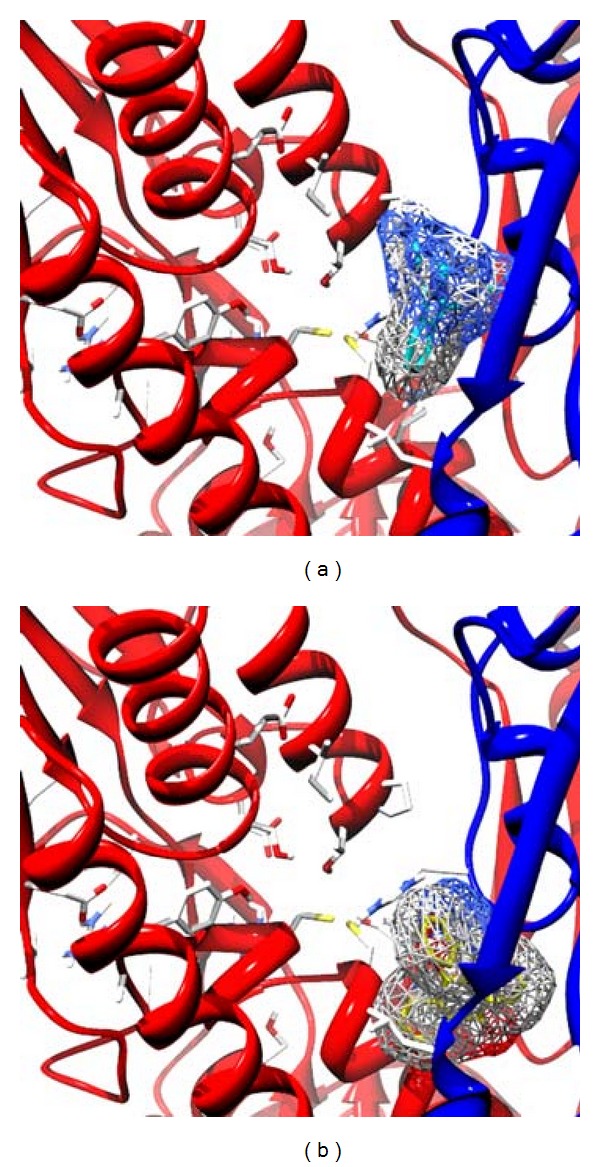
Binding Compound 31 (a) at the active site, the compound is lodged at the periphery of the active site, with potential hydrogen bonding interactions with Glu4666 and Glu467 making it a high-affinity interaction. Compound 47 (b) belonging to Cluster 4 binds to the additional hydrophobic patch with side chains extending towards the substrate-binding cleft.

**Figure 6:**
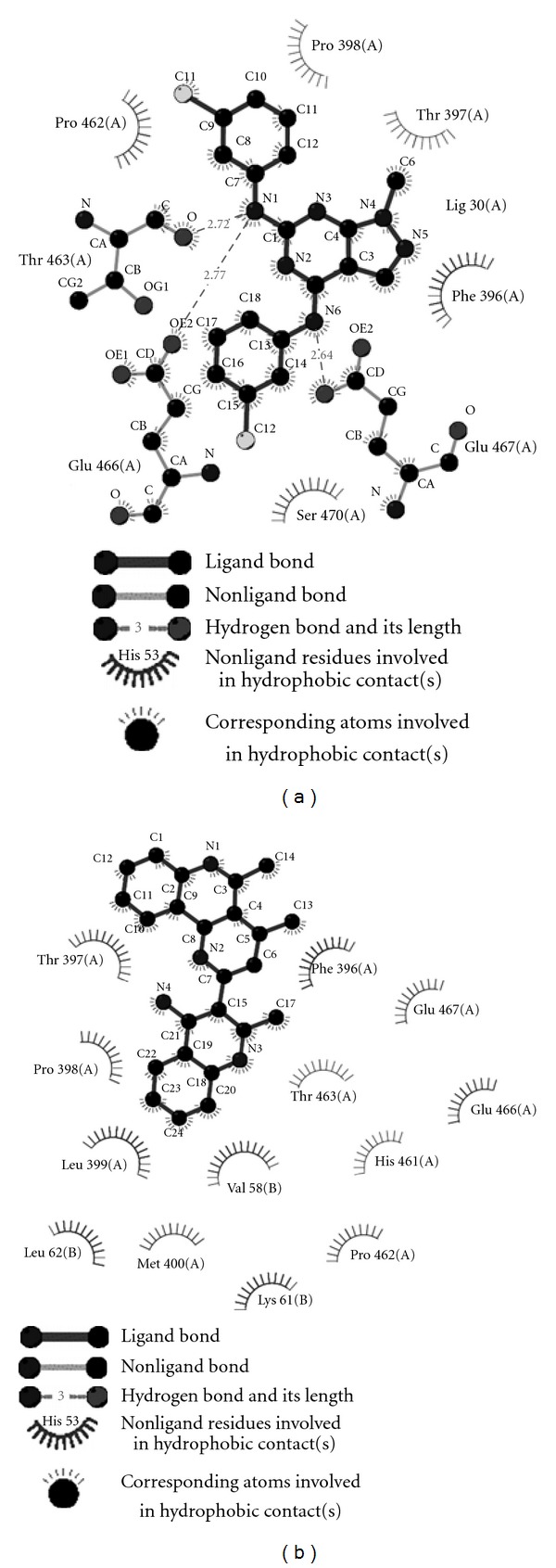
Compound 30 (a) of Cluster 3 in hydrogen bonding interaction with Glu466, Glu467, Thr463, Pro398, and Phe396 of the Z site is in hydrophobic contact with the inhibitor. Compound 35 (b) is in hydrophobic interaction with all the Z site residues, and there are other amino acids in non-boding interactions making it a high-affinity binding.

All the five inhibitors present in this cluster had hydrogen bonding potentials with Glu466 and Glu467; these residues form hydrogen bonds with of active site histidine (His461) orienting the side chain towards the hydride transfer site for reduction of T[S]_2_. Primarily, binding of inhibitors to this region is because of charge-based interaction, and they also have hydrophobic interactions with serine residues surrounding the active site making this conformation a highly favourable binding energy interaction. Inhibitors belonging to this cluster can be potential inhibitors of TR, by preventing the hydride transfer between T[S]_ 2_ and active site histidine. **Compound 31 **(Figure 5(a)) and **Compound 30 **(Figure 6(a)) in the cluster exhibit the conserved interaction pattern observed within SC-7; we hereby propose that charge-based interaction at this region with tricyclic moieties being lodged at the Z site can be developed as a rational ploy to selective inhibitors of TR.

### 3.4. Differential Binding Modes Exhibited by the Inhibitors at the Z Site

Cluster 4 comprises of larger number of conformations than any other cluster obtained through the self-organizing maps, five different sub-clusters were observed within the major cluster, the larger class of compounds present in this cluster are tricyclic compounds ranging from acridines to thiazenes, and few halogenated compounds. The higher affinity of tricyclic structures to the Z site amino acid residues and their favorable hydrophobic interaction with the inhibitors makes it a larger cluster, residues that are in conserved interaction within this cluster are Leu399, Pro398, and Phe396. Where Leu399 is a conservative substitution from GR among TR of all the Trypanosomatids, this additional hydrophobic region present in the vicinity of the substrate-binding site helps in stacking of tricyclic compounds, being the major class of inhibitor reports against TR. The side chains of inhibitors are in hydrogen bonding or non-bonding interaction with the residues of substrate-binding site.

In case of **Compound 1 **(SC-11), the inhibitor is in non bonding interaction with all the Z site residues and 10 other amino acids surrounding the active site; the stacking of pentacyclic structure between the hydrophobic patches makes it a highly favorable binding energy compound, no hydrogen bonding interaction was observed between the active site residues and this compound. **Compound 47** (SC-9) (Figure 5(b)) forms hydrogen bond with Thr463 of the active site, the tricyclic moiety of the compounds is docked against the hydrophobic patch of Z site and the side chains are extended towards the substrate-binding cleft. **Compound 35 **(Figure 6(b)) of SC-12, the single compound that was present in the subcluster, is in hydrophobic contact with Met400, Val58 and in charge-based interaction with Lys61 of the substrate-binding cleft along with conserved interaction of Cluster 4. The additional hydrophobic patch formed by the conserved substitution of Leu399 in TR of all Trypanosomatids can be utilized for selective designing of inhibitors towards the enzyme. Polycyclic compounds (tricyclic and pentacyclic) were found to have higher affinity towards the Z site; the protonated side chains of these classes of compounds can interfere with the binding of substrate to the active site by being in hydrogen bonding or non-bonding interactions with residues of substrate binding cleft, thereby inhibiting the reaction.

The results show diverse molecule sets binding with higher affinity to the active site of TR in four different conformations. In cluster 1, inhibitors are stacked between the two hydrophobic patches cluster 2 contains inhibitors which bind to the active in site an orientation which facilitates it to be in hydrogen bonding interaction with Z site amino acids and the protonated side chains to be in charge-based interaction with negatively charged region of the active site. Whereas in cluster 3 hydrogen bonding of inhibitors with Glu466 and Glu467 was observed and in cluster 4 inhibitors bind with higher affinity to the Z site, facilitated by hydrogen bonding and non-bonding interaction with surrounding amino acids. Although the active site of TR is comparatively larger when compared to active site of GR and small molecules bind to the active site in multiple orientations by clustering, it was evident the interactions are confined to four different regions in the active site, and more than one molecule of inhibitor can bind to the active site due to the multiple binding modes possible for any given inhibitor. We for the first time report selective inhibitors can be designed towards TR by combining the hydrophobic interaction of inhibitors with conserved Leu399 substitution and surrounding amino acids with charge-based interaction of Glu466 and Glu467 which can result in disruption of *in vitro* enzymatic conversion of T[S]_2_ to T[SH]_2_ by preventing the hydride transfer, the residues facilitate the orientation of His461 towards the hydride transfer region by hydrogen bonding to active site Histidine.

The hits representative of each cluster can be used for further development of specific inhibitors, and diverse binding modes explored by the method can be used for pharmacophore mapping in the process of designing more potent inhibitors against TR. The *in vitro* kinetics of each cluster vary depending upon their binding pattern, and contact based analysis of large chemical libraries can be performed to decrease the number of false positive hits obtained through the virtual screening process. For this purpose, true positive and false positive sets can be integrated into the virtual screening process.

The modeled binding modes provide insight into possible mode of binding that diverse set of compounds can attain at the active site of TR. Similar methodology can also be employed for other inhibitor screening processes, where contact-based ligand footprinting can be employed to discriminate between true positives and false positives. The screening process can also be supplemented with enzyme kinetic assays to validate true hits, which can be further modified for development into potential leads and drugs. Similar studies on the other enzymes of redox metabolism may be valuable towards novel drug discovery against leishmaniasis [24].

## 4. Conclusions

We report that alkaloid tomatidine and also few other dibenzothiaphenes, acridines can be potential inhibitors of TR. The differential binding mode of small molecules at the active site of TR has been clustered into four major clusters based upon ligand footprinting. The clusters have conserved interaction with Z site amino acids among them either as non-bonding interaction or hydrogen bonding interaction, reiterating the fact that this conservative substitution can be utilized for development of selective inhibitors towards TR. Some of the inhibitors here show that along with the chemical nature of the compounds net charge on the compound also plays a critical role in binding to the active site and also providing specificity towards TR. Binding strength of inhibitors and *in vitro* kinetics is dependent upon affinity and interaction of inhibitors towards the active site, so a contact-based clustering approach to classify inhibitors would provide effective segregation of different classes of inhibitors for a particular protein. The above-discussed method can be effectively used downstream of virtual screening processes or in combination with docking protocols to discriminate between different interaction patterns observed within a chemical library.

## Figures and Tables

**Table 1:** Structure and docking statistics of top 50 ranked structures. C, CL, E, A, and T indicate numbers of clusters, number of conformations within the selected cluster, binding energy of the selected conformation in kcal/mol, number of atoms in the inhibitor, and number of torsions, respectively.

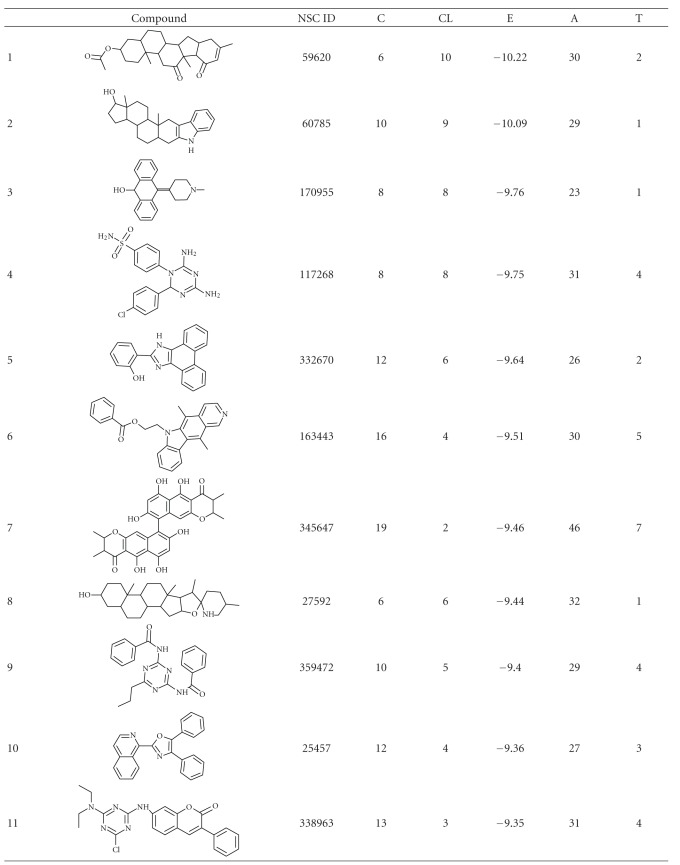 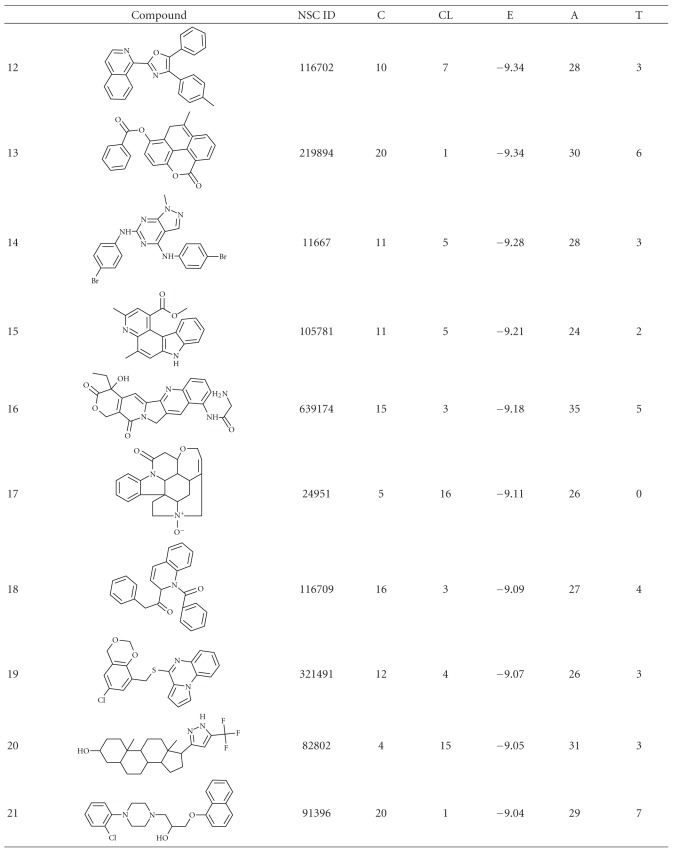 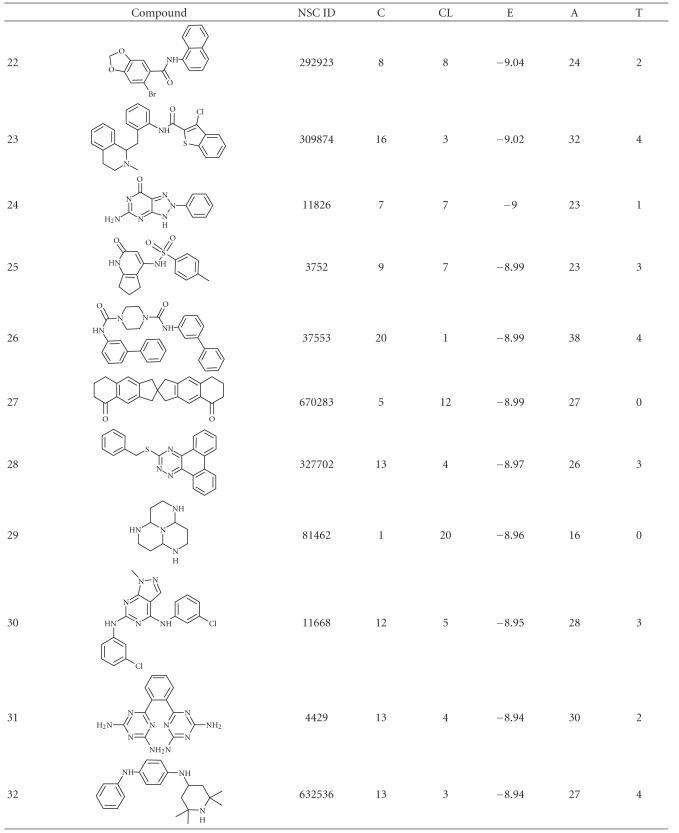 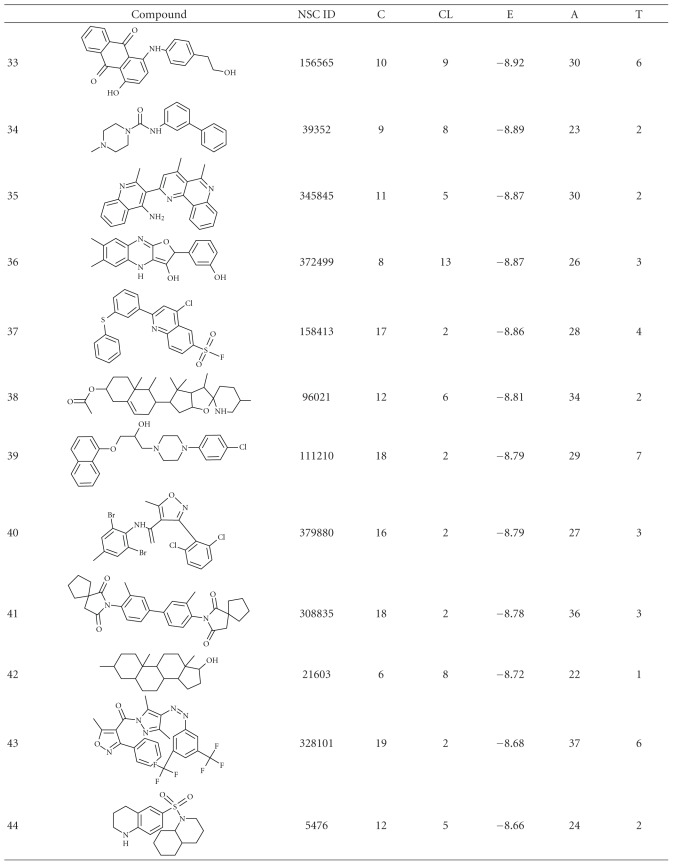 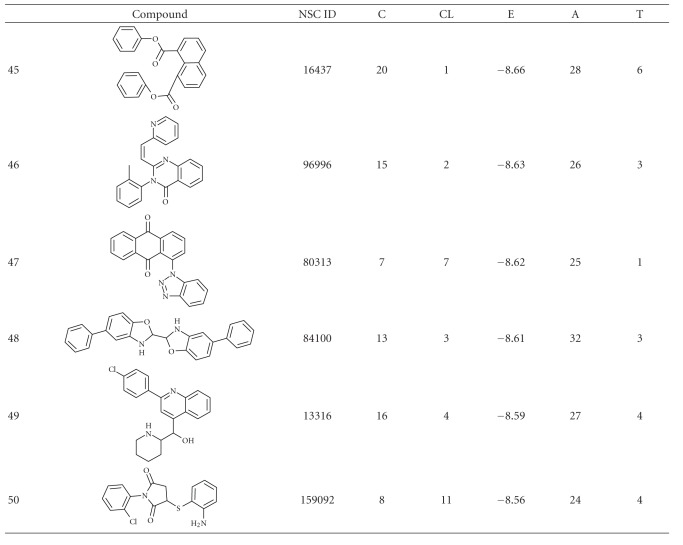

**Table 2:** Table showing conserved interaction of ligands with active site residues of TR within clusters.

S. no.	Cluster	Compounds present in the cluster*	Conserved interaction within the cluster (bonding and nonbonding interactions)
1	Cluster 1	SC-1 [Cpd3, Cpd8, Cpd9, Cpd16, Cpd38, Cpd43]	Leu399, Ser14, Glu18, Trp21, Tyr110, Met113, Cys52
		SC-2 [Cpd5, Cpd21, Cpd25]	
2	Cluster 2	SC-3[Cpd7, Cpd17, Cpd20, Cpd26, Cpd40, Cpd42]	His461, Leu399, Thr65, Glu466, Pro398, Thr396, Phe396, Pro398
		SC-4[Cpd2, Cpd15]	
		SC-5[Cpd24, Cpd37, Cpd48]	
		SC-6[Cpd6,Cpd23]	
3	Cluster 3	SC-7[Cpd14, Cpd29, Cpd30, Cpd31, Cpd49, Cpd32]	Glu466, Glu467, Thr463, Ser394
4	Cluster 4	SC-8[Cpd10, Cpd44, Cpd50]	Leu399, Pro398, Phe396,
		SC-9[Cpd12, Cpd19, Cpd27, Cpd28, Cpd33, Cpd34, Cpd36, Cpd46, Cpd47]	Met400, Lys61
		SC-10[Cpd22, Cpd45]	
		SC-11[Cpd1, Cpd4, Cpd11, Cpd13, Cpd18, Cpd39, Cpd41]	
		SC-12[Cpd35]	

*[ ] subclusters (SC) within major clusters are separated using [ ].
